# The Parallel Pandemic: A Systematic Review on the Effects of the COVID-19 Pandemic on OCD among Children and Adolescents

**DOI:** 10.3390/ijerph20237095

**Published:** 2023-11-22

**Authors:** Nasong A. Luginaah, Evans S. Batung, Bianca R. Ziegler, Daniel Amoak, John Paul Trudell, Godwin Arku, Isaac Luginaah

**Affiliations:** 1 Schulich School of Medicine and Dentistry, Western University, 1151 Richmond Street, London, ON N6A 3K7, Canada; nluginaah2027@meds.uwo.ca; 2 Department of Geography and Environment, Western University, 1151 Richmond Street, London, ON N6A 3K7, Canadailuginaa@uwo.ca (I.L.); 3 Department of Family Medicine, McMaster University, 1280 Main Street W, Hamilton, ON L8P 1H6, Canada

**Keywords:** COVID-19 pandemic, DSM-5, OCD, children, adolescent

## Abstract

The COVID-19 pandemic and the accompanying social changes severely impacted mental health globally. Children and adolescents may have been vulnerable to adverse mental health outcomes, especially obsessive-compulsive disorder (OCD), due to their underdeveloped resilience and coping skills stemming from their progressing physical and psychological development. Few studies have explored the parallels between the pandemic and OCD trends in this population. This systematic review aims to identify the impacts of COVID-19 on OCD among children and adolescents. Using the PRISMA guidelines, a systematic search of eight databases for studies that assessed OCD outcomes independently or as part of other psychiatric diagnoses during the COVID-19 pandemic was conducted. The search was limited to studies on humans and those written in English and published between January 2020 and May 2023. We identified 788 articles, out of which 71 were selected for a full-text review. Twenty-two papers were synthesized from 10 countries for the final analysis. We found that 77% of our studies suggested that the COVID-19 pandemic had a negative impact on OCD among children and adolescents. We also found a complex interplay of individual, household, and socio-structural factors associated with the aggravation of OCD. Conversely, a few studies revealed that the pandemic strengthened relationships and resilience. The findings of this study emphasize the need for mental health screening and support for this population, especially during pandemic periods.

## 1. Introduction

### 1.1. Obsessive-Compulsive Disorder among Children and Adolescents

Obsessive-compulsive disorder (OCD) is a long-lasting condition that causes significant functional impairment and negatively impacts quality of life [1]. OCD is characterized by anxiety-provoking intrusive thoughts, images, and obsessive behaviors to reduce anxiety or compulsions [1]. The DSM-5 (2010) reports that specific themes are commonly seen in the content of the obsessions and compulsions of patients. These themes include “(1) contamination obsessions and cleaning compulsions, (2) symmetry obsessions and repeating, ordering, and counting compulsions, (3) taboo thoughts (aggressive, sexual, or religious obsessions) and related compulsions, and (4) harm (fears of harm and checking compulsions)”.

While about two percent of the global population suffers from OCD [2], the disorder is more pronounced among children and adolescents, with a prevalence rate of 3% [3]. The impacts of OCD on children include negatively affecting their ability to initiate and maintain friendships as well as familial relationships [4]. Further, due to the significant amount of time spent focusing on these obsessions and compulsions, children may have difficulty concentrating and being on time, thus, impacting academic accomplishments and overall life course trajectory in later years. 

Throughout the COVID-19 pandemic, increased attention to behaviors associated with OCD, such as cleaning and decontamination, led to concern about an increased OCD prevalence. As suspected, OCD symptoms among children and youth have been reported to have increased during the pandemic. Due to the profound impact of OCD on functioning and quality of life, and the potential for public health measures and pandemics to exacerbate OCD, further research into this potential relationship is needed not only to plan a response to post-pandemic mental health challenges in society but also to plan for future pandemic responses. Consequently, this systematic review will summarize recent empirical work and provide areas for future research.

### 1.2. OCD and Associated Risk Factors

Earlier studies have highlighted that the higher incidences of OCD among children and adolescents may partly be driven by a complex interplay of pre-existing medical conditions and social, economic, and systemic factors [5,6,7]. Working within adolescent populations, study [8] 56% of the children with OCD also meet diagnostic criteria for at least one other comorbid condition, with the most common comorbidities being depression and anxiety disorders. Moreover, it has been hypothesized that gender may be one factor underlying differences in OCD symptoms. Previous studies among school children and youth found no gender differences in prevalence rates, age of onset, and/or symptom dimension [9,10], while those conducted on adolescents found a higher proportion of females with OCD [8].

In adult studies, gender has been associated with differential symptom presentation, course of illness, and comorbidity patterns [1,9,10]. Studies have found that contamination obsessions and cleaning compulsions are more frequent among females [9,10,11,12]. On the other hand, checking, sexual, exactness, and symmetry obsessions and compulsions have been found to be more common among males [9,10,11,12]. Females also have a higher severity of obsessions/compulsions among females, while comorbidity rates of social phobia and schizophrenia were higher in males [12]. Some studies also reported an earlier age of OCD onset in males [10,11].

### 1.3. OCD in the Context of COVID-19

Since the WHO declared COVID-19 as a pandemic in 2020, accentuating washing and disinfection in the process, the reports of OCD patients’ healthcare utilization increased as well [13]. Specifically, several studies demonstrate how the COVID-19 pandemic has been associated with adverse health outcomes and psychosocial distress in children and adolescents as well as increasing psychological difficulties including OCD [14,15,16,17]. Studies have shown that contamination obsessions and compulsive handwashing are among the most common OCD symptoms [18,19]. Further, according to [20], the contamination and cleaning–washing OCD subtype has been recognized to be associated with exaggerated threats and excessive responsibilities. During the pandemic, it was frequently emphasized that hand washing was one of the safest protection measures against infection.

Emphasis in the media on hygienic measures had the potential to heighten the risk perceptions of threat and responsibility in people with, or susceptible to, OCD and worsen their OCD symptoms [14,21]. Consequently, the increased accounts of the health problems associated with COVID-19 and recommended preventive measures, as presented in the media and by society, likely led to heighten the risk perceptions of threat and responsibility in people with, or susceptible to, OCD. It has been suggested that the social distancing and isolation measures that were put in place as part of efforts to curb the virus may have increased stress, aggravated feelings of loneliness, and impacted long-term health [22,23]. Given the severe impacts of societal changes that occurred during the pandemic, the effects on the mental health of young people cannot be overemphasized. As such, in children and adolescents with OCD, an increase in pandemic-driven anxiety and distress may have impacted their symptoms.

### 1.4. Aim of Study

While it is apparent that COVID-19 has profoundly affected the mental health of children and adolescents, evidence from most studies is not representative, making it difficult to draw significant population-based conclusions. More so, these studies present inconsistent and inconclusive findings, and little is known about the overall impact of COVID-19 on children and adolescents’ mental health, including OCD. Given the wide range of micro-level studies about children’s mental health in the context of COVID-19, we need a clear understanding of the most compelling factors that may be associated with OCD exacerbations among children and adolescents for effective policy making. To the best of our knowledge, except [3], who earlier reviewed six studies at the onset of the pandemic, no systematic review has addressed the impact of COVID-19 on OCD among children and adolescents. This systematic review aims to investigate the impact of the COVID-19 pandemic on children and adolescents with OCD and to identify the relative role of factors that may compound the experience of OCD symptoms. In accordance with the evidence-based practice searches, we constructed our research question following the population, exposure, outcome (PEO) formulation. Thus, the question that this study sought to answer is: what are the effects of COVID-19 on OCD outcomes among children and adolescents?

## 2. Methods

This systematic review was conducted based on the Preferred Reporting Items for Systematic Reviews and Meta-Analyses (PRISMA) guidelines. We registered the protocol of this systematic review in The International Prospective Register of Systematic Reviews (PROSPERO) database (CRD42021257890).

### 2.1. Search Strategy

We conducted a comprehensive literature search for published articles from the peer-reviewed databases: EMBASE, Web of Science, CINAHL, Psych INFO Ovid, Nursing and Allied Health, Scopus, Medline (Ovid), and PubMed. The initial search was conducted between 3 June 2021, and 7 June 2021. In terms of date ranges, we set 1 January 2019 as the lower limit since cases of COVID-19 began surfacing in various parts of the world during that year. For the upper limits, however, we used the date that the search was conducted, which varied depending on the database. Furthermore, we performed two supplementary searches to update our records. The first supplementary search occurred between 4 June and 6 June 2022, while the second was conducted between 7 June 2022 to 15 May 2023 using the same search terms (see Table 1). Regarding the date ranges for the supplementary searches, we set the start date as the last day of the previous search, while the end date was restricted to the day that we conducted the search. The authors generated the search terms with the assistance and support of a Research and Scholarly Communications Librarian from Western University in London, Ontario, Canada.

Search terms for the outcome of interest were decided based on the diagnoses included in the “Obsessive-Compulsive and Related Disorders” section of the *Diagnostics and Statistics Manual 5th edition* (DSM-5). This category of disorders constitutes OCD, body dysmorphic disorder, hoarding disorder, trichotillomania, excoriation disorder, substance/medication-induced obsessive-compulsive and related disorders, obsessive-compulsive and related disorders due to another medical condition, other specified obsessive-compulsive and related disorders, and unspecified obsessive-compulsive and related disorders. The DSM-5 posits that there is increasing evidence that these disorders are related to each other and have significant overlap; as a result, it is recommended that clinicians should screen for these conditions when one is present. Due to this grouping in the DSM-5 and this recommendation, our search terms included all these disorders within this DSM-5 category. 

A total of four independent reviewers (B.Z., E.B., N.L., and J.T.) were involved in the data search and data extraction. In the first literature search, each article was individually screened by reviewers B.Z., E.B., and N.L. Title and abstract screening was conducted first, followed by a full-text screening of the remaining articles based on inclusion and exclusion criteria. The results of the searches were limited to studies on humans and those written in English. After completing the literature search, results were exported from each database into Covidence—an online software that aids in organizing resources for systematic reviews by eliminating duplicate papers, supporting reviewers in identifying and resolving conflicts, and making the screening process more efficient.

Subsequent conflicts were resolved through a discussion between the three reviewers and a consultation with a fourth reviewer (J.T.). The authors repeated this process for the subsequent searches, although E.B. and N.L. individually screened each article in this phase.

### 2.2. Selection Criteria

Consistent with our PEO formulation, our inclusion and exclusion criteria were as follows.

Population: for this review, children or adolescents were defined as aged 0–17 (+364 days), in accordance with [24], and as such, studies that analyzed a population with an age range under 18 were included. In some studies, the pediatric population included slightly older adolescents (i.e., up to 21 or 22 years of age) due to [25] stating that a pediatric patient may be defined as up to 21 years of age; thus, in some cases, these articles were also included. Many studies had an adult population in addition to the pediatric population; in these instances, the paper was included if a subgroup analysis was conducted on the pediatric population.

Exposure: we selected articles that studied the effects of the COVID-19 virus.

Outcome: OCD symptomology was the outcome of interest. Studies that were solely focused on changes in treatment (i.e., telehealth interventions) for OCD were excluded as they were beyond the scope of this review.

Other considerations: to further narrow the precision of our search results, additional inclusion parameters were set, including limiting eligibility to studies that were carried out from 2019 until the upper limits of the search dates. We chose this time frame to encompass all literature in this field related to COVID-19, which emerged on the global stage in December 2019. Studies were not excluded based on the geographical region of the article due to the global impact of the COVID-19 pandemic. Studies that were not available in English were excluded.

### 2.3. Data Extraction

Three authors (B.Z., E.B., and N.L.) conducted the data extraction. Short-form data extracted from each study included the name of lead authors, year of publication, keywords, location of study, sample size, age of participants, mental health outcomes(s) of interest, and the pandemic and OCD correlation. Long-term data that was extracted also included the study design, key findings, and conclusions.

### 2.4. Quality Assessment

We evaluated all eligible studies using the National Heart, Lung, and Blood Institute (NIH) Study Quality Assessment Tools. We selected the NIH tool based on the study design of the articles, as different tools exist for various study designs. The assessment criteria for the studies included the clarity of the objective of the research, eligibility criteria, population of the study, sample size, outcomes that were analyzed, and the nature of the statistical analyses that were performed. Following the assessment, each study received a quality grade of either “good”, “fair”, or “poor”. Studies with 8+ “Yes” responses were deemed as “good”, whereas studies with scores between 4–7 “Yes” responses were deemed as “fair”. Studies scoring below 4, however, were considered as “poor”. Only studies that were deemed to be “good” or “fair” were included. In the quality assessment, each article was given a rating by two of three reviewers (N.L., E.B., and B.Z.), and disagreements were resolved through consultation with an additional author (J.T.).

### 2.5. Data Synthesis

We performed a narrative synthesis to synthesize the findings of the included studies. Due to the variety of eligible studies, narrative synthesis offers immense utility in collating and synthesizing evidence. Given the review’s focus on OCD, we relied on the summaries of key findings of studies to form a cross-study analysis that described the themes and differences in OCD outcomes along several dimensions, including gender, state of mental health, and other relevant characteristics. All included articles were inductively coded following the framework of [26] to allow themes and theories to emerge organically from the extracted data. Based on the thematic analysis, we examined the implication of COVID-19 on the OCD status of children and adolescents.

To this effect, neither a priori codes nor coding software were used. Following [27], manual coding was conducted by two authors (N.A.) and (E.B.) in three stages: open, axial, and selective coding. In open coding, each author read line-by-line, looking for similar themes, concepts, and relevant texts by reviewing all the articles and assigning initial codes to every paragraph or page. The codes that emerged from open coding were specific to the paragraphs or pages in the various articles. The authors then conducted axial coding, which involved looking for descriptive themes or concepts that emerged from the open coding. The two authors compared their codes and then agreed on a categorization of the initial codes into broader themes, including gender, parental influence, and access to services. All stages of coding and theme creation were conducted in consultation with and were reviewed by a third author (B.Z.), and discrepancies or disagreements were resolved through discussion between the three authors (N.A., E.B., and B.Z.). Given that the two authors conducted the coding in consultation with each other rather than separately, inter-coder reliability was not measured. Nevertheless, to ensure qualitative rigor through critical dialogue, the concept of “critical friends” [28] was used. Three of the authors (N.A., E.B. and B.Z.) and, when necessary, a fourth author (I.L.) offered their interpretations of the data and suggested codes and themes in all three stages of coding. The goal was to negotiate which codes and themes best supported the data and interpretation. Due to the inclusion of studies with varied designs and methodologies, the data collected was heterogeneous; therefore, meta-analysis could not be carried out.

## 3. Results

**Figure 1:**
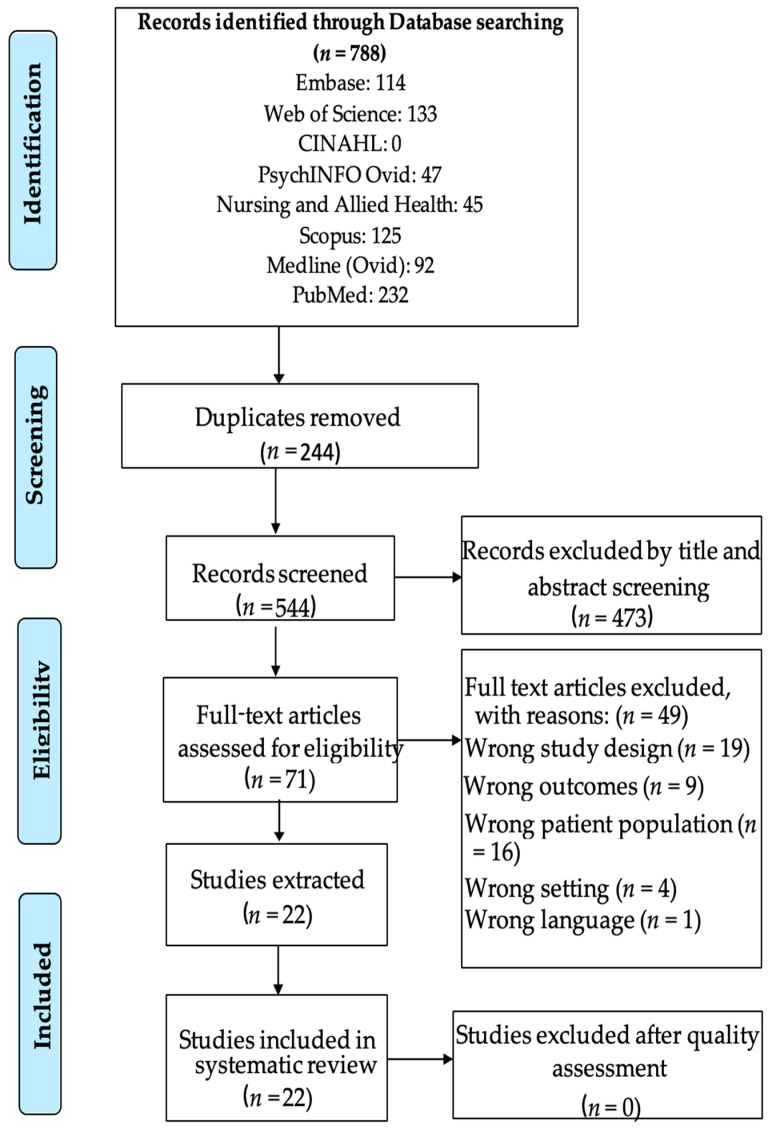
Prisma chart. Adapted From: Moher, D.; Liberati, A.; Tetzlaff, J.; Altman, D.G.; The PRISMA Group. Preferred reporting items for systematic reviews and meta-analyses: The PRISMA statement. *PLoS Med*. **2009**, *6*, e1000097. https://doi.org/10.1371/journal.pmed.1000097 [29].

Altogether, the databases yielded 788 articles; 244 of these articles were classified as duplicates and automatically removed from the Covidence software used. A total of 544 articles remained for the title and abstract screening, and 473 were excluded at the title and abstract screening stage by three reviewers (N.L., E.B., and B.Z.), and 71 went forward to a full-text review. Conflicts were settled by a fourth reviewer (J.T.). After employing the inclusion and exclusion criteria, 22 studies were deemed eligible and were included in the systematic review. See Figure 1 for the corresponding PRISMA chart and a brief description of each article in Table S1. 

### 3.1. Select Attributes of Included Studies

#### 3.1.1. Year of Article Publication

The included studies (see Table S1 in the supplementary material section) were published between 2020 and 2023, with the most articles (5) being published in 2022. Both 2020 and 2021 had an equal number of titles (5), while 2023 had the least number (4). This low number likely suggests that although the interplay of COVID-19 and OCD among vulnerable groups is gaining traction, empirical studies are still very limited.

#### 3.1.2. Spatial Distribution

**Figure 2:**
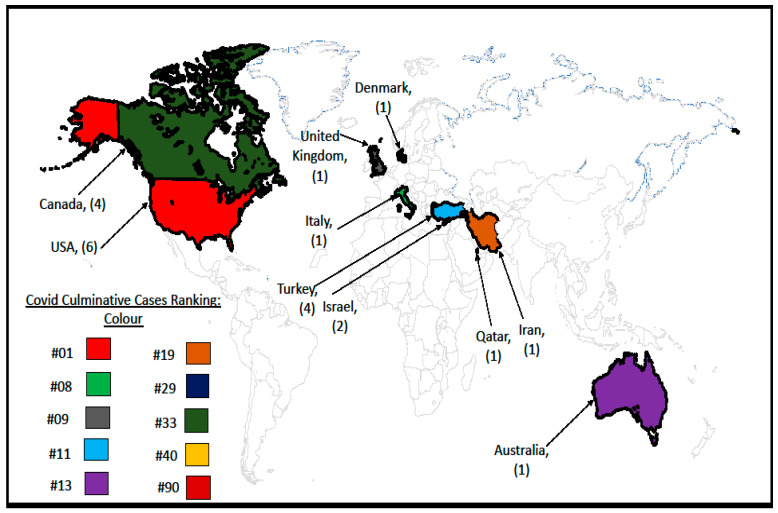
Geographic distribution of selected articles (*n* = 22). Accessed from WHO Coronavirus (COVID-19 Dashboard). https://covid19.who.int/table, accessed on 17 September 2022. World Health Organization COVID-19 Dashboard (2022). WHO COVID-19 Dashboard https://covid19.who.int/, accessed on 17 September 2022.

Further, Figure 2 shows the geographical spread of the articles included in this systematic review and the number of studies associated with each place. Although the pandemic was global in nature, the literature on its effect on OCD among children and adolescents is relatively sparse and predominantly studied in the Global North. Overall, North America (10) and Asia (8) had the highest number of articles, followed by Europe (3) and then Australia (1). We also included each country’s “number of COVID-19 case ranking” in Figure 2 at the time of the data extraction, based on data from the World Health Organization (WHO).

#### 3.1.3. Clinical Diagnosis of Sample

Most articles (82%) studied populations with diverse clinical diagnoses. These included children and adolescents diagnosed with autism [5], OCD [30,31,32,33,34,35,36], Tourette’s syndrome (TS), and general mental health and behavioral disorders [7,37,38,39,40].

Some authors also focused on groups with multiple diagnoses. For instance, study [41] studied children diagnosed with chronic tic disorders (CTD), OCD, and tics with OCD. Similarly, study [42] also focused on children with PANS/PANDAS. Pediatric autoimmune disorder associated with streptococcal infections (PANDAS) refers to the abrupt and significant onset of OCD, tics, or restricted eating following a group A streptococcus infection (GAS) [43]. Pediatric acute-onset neuropsychiatric syndrome (PANS) describes the acute onset of OCD and/or severe restricted eating, accompanied by at least two ancillary criteria related to cognitive, behavioral, or neurological symptoms [44]. Study [45] also narrowly studied patients with DSM-5-related psychiatric complaints. Conversely, the study [4] excluded children if they had psychological or physical comorbidities before the beginning of the COVID-19 pandemic.

#### 3.1.4. Age of Sample

Regarding age, 68% of the articles studied children and adolescents aged 18 and under. But as mentioned earlier, we also included select studies with a slightly older population. For example, study [34] focused on patients up to 21 years old, while a little over a tenth (14.8%) of the population of study [46] were older than 18 years. Study [31] also limited their study to young people aged 13 to 19. In studies [30,47] researchers also worked with pediatric samples up to 18 years old; Similarly, studies [48,49] also researched pandemic-induced OCD trends among K-12 students at a developmental research school, which made it highly likely that 18-year-olds were included in the study.

#### 3.1.5. Gender of Sample

Regarding gender distributions, there were no gender-specific studies. However, the sample in most studies (95%) were predominantly female [31,32,33,34,38,40,45,46,47,48,50]. Study [39] did not disclose the gender distribution of their study sample.

### 3.2. Quality Assessment

The quality appraisals of the included studies (*n* = 22) are summarized in Table S2. Out of the 22 studies included, 18 had a quality that was rated as good (score of 8 and above). The remaining four studies were rated as fair, all with varying risk of bias that was still slight enough to be suitable for inclusion.

Table S2: Quality assessment score of selected articles (added as a Supplementary File).

### 3.3. Key Findings

As shown in Table S1, the relationships between the COVID-19 pandemic and OCD among children and adolescents were mixed. That notwithstanding, an overwhelming majority of the included studies (*n* = 17; 77%) reported that the COVID-19 pandemic had an adverse impact on OCD among children and adolescents [4,5,7,32,33,34,36,38,39,40,41,42,45,46,48,49,50]. A few studies (*n* = 3, 17%) found mixed relationships [31,37,47], and two (*n* = 2, 9%) found no influence of the pandemic on OCD among children and adolescents [30,51]. Despite these mixed relationships, several crucial factors emerged from the included studies. We grouped these factors into three broad themes: individual, household relations and familial dynamics, and socio-structural factors (see Table 2). Of note, some studies identified multiple factors that impacted OCD; as such, the total number of studies reported in the “Article References” category on Table 2 exceeded 22.

#### 3.3.1. Individual-Level Factors

##### Comorbidities or Prior Diagnosis

Individual-level factors were the most mentioned theme (27) influencing the relationship between the pandemic and OCD among children and adolescents. In this theme, comorbidities or prior diagnosis were the most common factors influencing OCD in 10 studies (Table S2). Overall, children and adolescents who were diagnosed with other conditions, including prior COVID-19 infection [42], pre-COVID psychiatric diagnosis of OCD [32,38,39], presentation of aggressive and sexual symptoms [34], and pre-COVID psychiatric diagnosis of comorbid mental health and autism spectrum disorder/developmental delay [5,37,42,50] experienced higher rates of obsessions and worsening of OCD symptoms during the pandemic. In study [49], a three-point longitudinal study, participants that were previously identified as at risk for OCD in any of the earlier two time points were associated with risk of OCD in the third time point during the pandemic. Study [41], however, found that COVID-19 had a negative impact on OCD symptoms with or without comorbidities.

##### Age

Age was also found to impact OCD outcomes among children and adolescents in four studies; some of the findings were contradictory, however. Three of the four studies found a significant correlation between OCD symptoms worsening among older adolescents when compared to their younger counterparts [32,37,50]. On the contrary, study [34] reported higher levels of worsening OCD symptoms among younger participants, as well as in those with earlier age of onset compared to their older counterparts. 

##### Gender and Sexual Orientation

Similarly, gender impacted OCD outcomes among children and adolescents in four studies. In general, female participants had a higher prevalence of OCD-related symptoms [31,38,50] and were at a higher risk of OCD symptoms during the pandemic [48]. Furthermore, study [50] also found that those identifying as gender non-binary and LGBTQ2S+ were significantly at risk of OCD and other psychiatric outcomes across all age groups, including children and youth.

##### Pandemic-Related Fears and Stress

Three studies cited the role of pandemic-related fears and stress in shaping OCD outcomes among children and adolescents. Overall, pandemic-related fears and stress in the form of increased anxiety, worrying, and COVID-19 mental preoccupation were significantly associated with worsening OCD symptoms [7,32,34].

##### Isolation

Isolation influenced OCD outcomes in two studies. Both studies found significant levels of OCD symptom deterioration among participants who experienced social isolation from COVID-19 lockdown measures at the height of the pandemic when compared to the periods when the pandemic was not as severe [37,39].

##### Race, Educational Attainment, and Personal Beliefs and Attitudes

Despite being the least cited factors in this theme, with only one mention each, the roles of race, education, and personal beliefs and attitudes cannot be understated. Racial background significantly increased the risk of OCD among non-White participants compared to their white counterparts [49]. The prevalence of White students, Black students, Hispanic students, and Multiracial/Other participants were 22.8%, 50.0%, 42.9%, and 100%, respectively.

Regarding education, study [48] found that primary school and middle school students were more likely to be at risk for OCD and anxiety-related symptoms compared to high school students. Further, primary school students were even more likely to be at “high risk” than their high school counterparts. Study [47] also revealed that participants who believed COVID-19 to be a severe illness were associated with OCD-related behaviors such as frequent disinfecting. Similarly, compared to participants who endorsed greater social trust and social responsibility, those with “greater self-interest values” (self-interest values relate to how youth rated the importance of putting their own needs before the needs of others and doing what they want regardless of what other people might want) engaged in greater OCD-related behaviors such as hoarding.

#### 3.3.2. Household Relations and Familial Dynamics

##### Socio-Economic Status (SES)

At the household level, SES was one of the most cited factors that impacted OCD outcomes among children and adolescents, with four studies reporting on this factor. All four studies revealed a significantly adverse effect of poor SES on OCD prevalence and worsening of symptoms [5,37,48,50]. We must highlight the nuances in conceptualizing “SES” across these studies. Study [48] measured SES as “income loss”, study [5] estimated it as “greater economic concerns and material deprivation (material deprivation is defined as the inability for individuals or households to afford those consumption goods and activities that are typical in a society at a given point in time, irrespective of people’s preferences with respect to these items (https://stats.oecd.org/glossary/detail.asp?ID=7326#:~:text=Material%20deprivation%20refers%20to%20the,with%20respect%20to%20these%20items, accessed on 8 July 2023))”, while [50] captured “poverty” as self-reported income under CAD 75,000 and/or food insecurity.

##### Death or Diagnosis of Relatives and Family Members from COVID-19 and Other Illnesses

Diagnosis of relatives and family members with COVID-19 was also a highly cited (4) factor shaping OCD outcomes among children and adolescents. All the studies unequivocally reported a significant association between participants’ adverse OCD outcomes and diagnoses of relatives and family members [4,34,36,49]. OCD was associated with participants who lost a family member due to COVID-19 [50]. Study [36] also found a significant association between participants’ OCD severity score and diagnosis of COVID-19 in someone familiar. Relatedly, study [4] uncovered a significant correlation between OCD symptoms and the presence of a family member diagnosed with COVID-19 and a frontline worker in the family. Furthermore, study [34] identified an increase in OCD aggressive and sexual symptoms among participants with a family history of ADHD.

##### Parental Influence

Beyond family-related deaths and diagnosis, the parental inclination toward COVID-19—primarily through their beliefs, mental health status, pandemic worries, and practices—were also crucial determinants of OCD outcomes among children and adolescents. The specific impacts of parental influence, however, were mixed. For instance, two of the three studies that cited this factor found parents’ worsening mental health and COVID-related stress negatively impacted their children’s mental health, including OCD [5,39].

A third study, however, interestingly found that participants whose parents believed vaccinating their child was not crucial for the health of others in the community were less likely to present with symptoms of OCD [49].

##### Increased Hygiene Protocols

Three articles reported the influence of hygiene protocols on OCD outcomes among children and adolescents. Study [31] found that participants with washing compulsions were associated with the highest prevalence of OCD symptoms.

Study [32] reported increased disorder severity, contamination obsessions, and compulsions like cleaning symptoms. Likewise, study [36] found a significant increase in the frequency of contamination obsessions and cleaning/washing compulsions during the pandemic.

#### 3.3.3. Socio-Structural Factors

The changes that took place at the broader societal level in an effort to ensure public safety, educate people, and mitigate COVID-19 infections were also cited in six studies to have adversely influenced OCD outcomes among children and adolescents.

##### Systemic Changes and Loss of Services

Three studies referenced systemic changes, including the loss of some essential services shaping OCD. For instance, all three studies found that the closure of schools, especially for students with special needs, and medical services significantly contributed to the worsening of OCD among their participants [5,39,50].

##### COVID-19 Media Exposure and Preoccupation

Another vital dimension of the dramatic changes that characterized society, the saturation of media outlets with COVID-related information, was cited in two studies as playing a critical role in shaping OCD outcomes among children and adolescents. Study [36] reported a significantly positive relationship between worsening OCD symptoms and talking or searching in their social environment about COVID-19.

In a separate analysis [41], they studied children diagnosed with three unique mental health outcomes—chronic tic disorders (CTD), OCD only, and Tics + OCD—and found that among the three subgroups, 50% of the OCD-only group used screens for more than 8 h per day during the weekday. Additionally, 33% used screens for more than 8 h per day on the weekend, which is higher than the screen times of the Tics + OCD group on both weekdays (50% used screens for approximately 3–8 h) and weekends (25% used screens for more than 8 h). Unsurprisingly, study [41] also reported that compared to the other groups, the OCD-only group experienced worse frustration and anger and rated their home life similarly worse.

##### Social Support

Although social support was the least cited factor in this theme, its impact was equally relevant. Study [46] found that a shorter time dedicated to school activities during the lockdown was associated with worsening obsessions and compulsions as participants could no longer interact with their peers who were integral to their social networks.

### 3.4. Forging Resilience in the Pandemic

Surprisingly, five studies [30,37,41,42,51] reported outcomes that were contrary to the aforementioned trends. Despite the largely adverse mental health outcomes associated with the COVID-19 pandemic, some participants’ OCD symptoms did not worsen, while others emerged from the pandemic with more resiliency, better mental health outcomes, and a generally positive outlook on life compared to pre-pandemic times. In Israel, for example, study [51] found that OCD symptoms did not worsen during the pandemic. On the contrary, a higher proportion of children with OCD reported improved functioning than those reporting a deterioration in functioning.

Study [30] reported similar trends from their multi-point (2 and 6 months) follow-up study of OCD patients who were actively undergoing exposure and response prevention (ERP) combined with pharmacological treatment. From the 2-month follow-up, a significantly higher proportion of the participants reported that COVID-19 did not affect their OCD when compared to those who gave other responses (probably not; probably yes). The results at the 6-month follow-up mark showed a similar trend as the number of OCD patients that reported that COVID-19 did not affect their OCD was significantly higher than the numbers providing other answers (probably not; probably yes; and yes).

Working with caregivers of children with OCD and other psychiatric diagnoses, study [42] shared that most of their respondents indicated that despite the pandemic’s strain on social relations, it positively influenced their relationship with their children. Similarly, study [41] found that most of their study participants reported no change in their relationship with their parents. Among those reporting changes, however, positive reports outweighed negative ones.

## 4. Discussion

This systematic review aimed to assess the impact of COVID-19 on OCD among children and adolescents. Based on the studies we reviewed, 77% (*n* = 17) reported that the COVID-19 pandemic had an adverse impact on OCD among children and adolescents. While most of the pandemic’s effects were largely negative, we also uncovered some positive outcomes in a few instances. Our findings further attribute this result to multiple individual, household, and broader societal factors. We further elaborate on these in the sections that follow.

### 4.1. Individual Level

At the individual level, we found that comorbidities or prior diagnosis, age, gender, sexual orientation, race, pandemic-related fears and stress, isolation, education attainment, and personal beliefs and attitudes contributed to increased OCD stress among children and adolescents amid COVID-19. A notable number of studies [5,32,34,37,39,41,42,49,50] in our review found that the worsening of OCD symptoms was significantly associated with comorbidities or prior diagnosis among children and adolescents, especially those that are psychiatric in nature [52,53]. When epidemics and other social disruptions arise, people with pre-existing psychiatric conditions are usually most vulnerable and differentially impacted [54]. A primary reason is that mental health disorders can increase their exposure via little awareness, increasing the risk of infections [53]. The highly emotional and unpredictable nature of the COVID-19 pandemic also brought waves of stress, stigma, medication defaults, fear, anxiety, and depression, which can trigger relapses or worsening of these pre-existing psychiatric symptoms [53,54]. An analysis of a cluster of 50 cases of COVID-19 among inpatients in one psychiatric hospital in Wuhan during the first wave of the pandemic highlighted the role of mental disorders in coronavirus transmission [53]. This finding is consistent with previous studies showing that pre-existing conditions can create unique mental health challenges for children and adolescents, such as increasing the likelihood of OCD individuals expressing severe and elevated symptoms [52,53].

Furthermore, since comorbid individuals are at a greater risk of experiencing the COVID-19 virus’ more severe symptoms, their fear and anxiety about the possibility of complications following contracting the virus may cause them to excessively practice precautions related to COVID-19, such as frequent handwashing, which may also trigger OCD symptoms in comorbid children and adolescents. Therefore, such conditions may likely significantly impact the severity of OCD symptoms in children and adolescents and explain the large prevalence of studies included in our review that cited comorbidities as a factor [55,56].

We also found that age was significantly associated with the worsening of OCD symptoms [32,37,50]. This relationship may be explained by the fact that mental illness, including OCD, generally emerges and peaks around 14 to 25 years. This window is a particularly sensitive period in the human physiological development cycle [57]. Furthermore, this age group also has an underlying vulnerability, even in pre-pandemic times. Earlier research highlights this phase as the most fragile in terms of lapse of service between child and adult mental health systems [58]. Therefore, considering the social turmoil that accompanied the pandemic, with school and job closures and shut down of most socially interactive spaces, it is reasonable that people in this age group were disproportionately affected [59]. This finding is consistent with other studies that have reported similar results [50,55,56].

The results also indicated that gender was also associated with increased OCD symptoms in children and adolescents [31,38,48,50]. Potential reasons for these gender-based differences may be partly explained by cultural norms and gender roles that may influence women’s affinity with cleanliness and good health, which may influence OCD symptoms among women [60]. Females, in general, are also more susceptible to stressors. Hence, the prolonged exposure to the multiple waves of the pandemic may have uniquely increased their vulnerability [50]. Furthermore, societal influences, including culture and traditional undertones of masculinities, may affect the reporting of OCD symptoms among men [60]. Additionally, the findings in this review on gender may also be due to differences in methods of measurement that were used by the different studies reported. For instance, study [61] suggested that OCD symptoms are best measured using dimensional scales of each symptom dimension. However, we found that some of the studies included in this systematic review used a variety of measures to assess the presence of OCD in children and adolescents, which may impact the findings which are consistent with a recent meta-analysis of global studies that revealed that women are about 1.6 times more likely than men to experience OCD [60].

Relatedly, children and adolescents identifying as LGBTQ2S+ and non-binary were similarly adversely impacted by the pandemic [50]. Sexual obsessions include recurrent doubts about whether one is gay or straight, fears of homosexual inclination, and being perceived as homosexual by others [62]. A plausible explanation of this outcome can be attributed to protracted periods of lockdowns and isolation, which increases the complexity of experiences for a group still grappling with social exclusion and marginalization [63]. These adverse experiences may even worsen for those locked down with unsupportive family members [64] and other hostile contexts [65]. Gender non-binary individuals specifically have been found to demonstrate healthcare avoidance during the pandemic, which might have increased the severity of psychiatric diagnoses, including OCD [66]. The link between sexual orientation, primarily expressed through sexual obsessions, has long been established in the literature [50,67,68].

We also noted that race and lower levels of academic attainment are associated with poor mental health outcomes, including OCD [48,49]. In this regard, the negative impacts of the pandemic can be conceived as a neutral external threat that was only given shape and direction by historical patterns of structural social disenfranchisement [69]. Unfortunately, this is inextricably linked to racial or ethnic undertones that underlie inequalities in health and income, which are significant determinants of health that shape exposure to the virus, medical care accessibility, and adherence to safety—safe spacing within the household and frequent testing [70]. Our findings on race largely concur with a growing body of research highlighting the pandemic’s disproportionate impact on minority groups [49,70,71]

In terms of the role of education, the observed difference in the studies included in our review [47] might be caused by children and adolescents with lower educational levels not fully comprehending COVID-19 guidelines and best practices, which may exacerbate their exposure, vulnerability, and psychosocial outcomes [48,55,72]. Given that younger children primarily do not accrue higher education attainments, it is not surprising that age was associated with the experience of OCD symptoms. Younger children may not benefit from effective COVID-19 communications from parents, mass media, and at school due to their limited capacity to comprehend these guidelines, unlike their older counterparts [73].

Another noteworthy finding of our study was that children and adolescents who reported more pandemic-related fears and stress had worsening OCD outcomes [47]. As [74] pointed out, the COVID-19 pandemic has resulted in severe levels of stress and fear among the general population, including children. This pandemic fear and anxiety experienced by children were further exacerbated by lockdowns, which deprived them of essential opportunities to interact with peers and friends. The combination of these factors resulted in low mental health for children, which culminated in elevated levels of OCD deterioration. In keeping with this argument, we found a linkage between isolation and aggravation of OCD symptoms among children and adolescents [37,39].

The sense of isolation arising from the need to be around others made nearly impossible during stringent COVID-19-related public health measures has been particularly destructive to individuals’ mental health [75].

### 4.2. Household Relations and Familial Dynamic

Our finding at the household level indicate that income and material deprivation are significantly associated with OCD symptom severity and worsening among children and adolescents. This relationship reiterates the importance of broader contextualization of diseases [5,37,48,50]. Staying at home, for instance, alongside the disruption of employment, threatened household financial stability as the lockdowns generally accelerated the expenditure of disposable income that could play critical roles in other protective investments. Even in pre-pandemic times, financial instability [52,75] and diverse poor socioeconomic outcomes have been common among people with mental illnesses [69,76]. In an earlier analysis of parental education on child mental health problems, the relationship was only significant among children within the lowest GDP per capita category [76]. This finding bolsters the linkages between precarity, mental health exacerbation, and penury. For OCD development, particularly, a low socioeconomic level has been identified as a risk factor [77]. In Denmark, children born into lower socioeconomic households have been found to have higher OCD risks than children born into higher socioeconomic homes, especially girls [34].

In contrast, children in higher-income households with OCD were less likely to experience increased symptoms [5,48]. In the context of COVID-19, children may be at a higher risk of OCD due to lower socioeconomic status, larger family composition, overcrowding, and poor access to healthcare. Previous studies have found that crowded homes, which result from low socioeconomic status, are linked to OCD among children [78].

Furthermore, the lockdown mandates also meant that children and adolescents had to spend more time with family and close relatives, which may have deepened the effects of diverse child–parent interactions and increased their exposure to new aspects of family life. Our finding that household relations and familial dynamics are associated with increased OCD-related manifestations and stress among children and adolescents during COVID-19 further tightens these rather loose assumptions. Notably, parental influence, such as having a relative diagnosed with COVID-19, and household economic status contributed to increased OCD symptoms among children and adolescents [5,39,48]. Interestingly, evidence suggests that the OCD symptoms of children and adolescents were worsened when authority figures within their households exhibited pandemic-related fears. This effect is likely because these authority figures, including parents and guardians, play a significant role in managing a child’s stress levels [48]. Moreover, when relatives are infected with the virus, the proximity of the infected person to children and adolescents may cause them to take extra precautions not to become infected [4,34,36,49]. To this effect, OCD behaviors, including excessive handwashing and disinfecting, may be triggered or exacerbated. During COVID-19 lockdowns, children and adolescents living with a family member who was infected with the virus experienced decreased healthcare access, which compounded their stress and anxiety [79]. These factors, taken together, illustrate the large effect household dynamics during the COVID-19 pandemic had on children and adolescents with OCD.

### 4.3. Socio-Structural

When considering our findings at the socio-structural level, a major factor is the systemic changes and loss of services [5,39,50]. At the societal level, the drastic changes brought by the pandemic were also crucial in shaping OCD outcomes. Study [80] cautioned that environmental factors are at least as important as genetic factors for developing obsessive-compulsive symptoms. They must, therefore, be thoroughly considered in the health discourse. During the lockdowns, which significantly increased screen times, children and adolescents were constantly exposed to COVID-19 messages in the media, which may have had deleterious effects on them [36,41]. Given that the priority of public health officials and media outlets focused on the outbreak’s biological and physical implications, its mental health implications were very much neglected. Indeed, study [81] found that patients with worsening OCD showed higher rates of Internet checking for health reassurance. This observation is consistent with OCD-related intolerance of uncertainty along with frequent and excessive online health searches [14,81].

In addition, as children and adolescents become more anxious, depressed, and adversely affected by avoidance and extreme preventive behaviors, their conditions may worsen [53]. Unsurprisingly, study [31,32,36] also found that the highest rates of OCD compulsions were those in the washing and contamination category.

Another crucial social change that shaped OCD outcomes relates to the loss of essential services and social support systems. Sweeping stay-at-home requirements severely affected healthcare access and delivery, which interrupted the progressions of psychiatric care [82,83]. Without any rapid, proactive, and efficient transition plan, this treatment hiatus inevitably increased the uncertainty, inaccessibility and risk of relapses or worsening of existing mental health conditions during the COVID-19 pandemic [81]. According to [84], many OCD patients might have been reluctant to ask for assistance due to stigma or lack of awareness of what “excessive” behavior means regarding behaviors such as washing compulsions.

Similarly, the closure of schools and other controlled social settings that double as the support systems of children and adolescents was also a crucial driver of OCD outcomes [46]. As [52,75] highlighted, social networks are common among people with mental illnesses. Thus, restricted social connectivity during COVID-19 significantly stressed these individuals. Looking at the broader picture, this is unsurprising considering our earlier finding that older adolescents, usually of school-going age, had more adverse OCD outcomes [57].

## 5. Strength and Limitations

A major strength of this study is the number of studies that were included in the review. A prior study conducted by study [3] on a similar topic comprised only six studies. As such, this study builds on earlier evidence to present a more comprehensive description of current literature. Additionally, using standardized techniques to assess the quality of included studies (i.e., PRISMA) aids in detecting potential sources of biases and assessing the overall quality of the evidence provided. Employing a rigid and standardized approach, with clearly defined inclusion and exclusion criteria for data extraction, aided in reducing errors and allowed for the meaningful comparison of the extent of OCID-19 impacts on OCD among children and adolescents. Moreover, 18 of the studies included in our study had a quality assessment score of good. The high quality of the included articles adds to the validity of this review.

Despite the significance of our results, they must be interpreted against some limitations. First, the OCD variable in a few of the studies was constructed from a combination with other psychiatric diagnoses and, thus, limits the OCD-specific generalizability of findings from those studies. Specifically, this is a potential source of bias as not all counts of OCD diagnosis may be conclusively attributed to OCD. Another limitation of our study may stem from the intrinsic bias of the results of each study, including the influence of confounding factors. Nevertheless, some authors deemed this composite measure of OCD necessary to reduce subject fatigue. Additionally, there was sample attrition in some of the follow-up studies, which can introduce a certain level of bias. Also, most of the studies in this review were cross-sectional studies conducted during the pandemic and may not reflect the long-term effects of the COVID-19 pandemic on young people with OCD.

Next, some studies relied on telephone or online interviews due to home confinement for this age group during lockdown, and this may have affected the diagnosis of symptoms. Also, the studies used different measurement scales, which can influence the reported outcomes. Finally, the worsening of OCD symptoms may also be a natural course of an individual’s disorder, which may or may not be attributable to COVID-19. Longitudinal studies with larger samples are needed to elucidate further the impact of COVID-19 on the mental health of children and adolescents. Available longitudinal had relatively short follow-up durations due to the prompt nature of much of the research regarding the pandemic. As such, this is a potential blind spot in the literature and in this current study.

## 6. Conclusions

The study systematically reviewed the effects of the COVID-19 pandemic on OCD outcomes among children and adolescents. Our findings show that the COVID-19 pandemic had a negative impact on OCD among children and adolescents, both in terms of prevalence and symptom deterioration. Overall, the findings in the review showed several factors that influenced OCD symptoms during the COVID-19 pandemic. With regards to gender, female adolescents were more likely to report OCD symptoms during the pandemic, thereby highlighting the need to be proactive when dealing with young adult females in the context of a pandemic. Socioeconomic inequalities also influenced OCD-related symptoms, as participants who were of low SES were associated with negative outcomes. Importantly, knowing a family member had COVID-19 resulted in increased reporting of OCD symptom reporting. Furthermore, increased COVID-19 prevention protocols, such as frequent washing of hands, which precipitated washing compulsions in some cases, resulted in OCD symptoms.

Based on our findings, it is imperative that during pandemics, as public health experts focus on reducing the spread of infections, it is essential that they also focus on addressing the potential psychosocial needs of vulnerable populations, especially children and adolescents. Ignoring the nuanced impact of the disease or any other social turbulence on this population or similarly vulnerable groups will not only hinder efforts to minimize adverse effects but will also promote already existing health inequalities. Regarding mental healthcare services, this finding emphasizes the need for relevant stakeholders to implement rapid response systems that will sustain support systems for people with mental health concerns [52].

## 7. Recommendations

Notwithstanding these limitations, our findings shed some light on important considerations for healthcare practitioners. First, given the immense impact of COVID-19 on children, there is a need for more research to improve our understanding of intra-group vulnerabilities that constitute children and adolescents, especially children in low-income households and those from other marginalized backgrounds. Most studies we included in this review were conducted in high-income countries; therefore, future research must include countries in the Global South to ensure a more comprehensive understanding of the impact of COVID-19 and subsequent outbreaks.

It is also crucial to rapidly address the gaps in medical treatment between transitioning in-person services to virtual delivery. As such, having a robust system of video-consults and online psychotherapy measures to support young people with OCD is a viable option [31].

Study [48] also suggested that within the COVID-19 pandemic, schools should prioritize mental health interventions that target younger female students and children of families with income loss. Although limiting the spread of COVID-19 through school closures was necessary, it likely exacerbated adverse psychosocial health outcomes in children [84]. Therefore, we recommend that during social disruptions, school administrators and public health workers should be proactive in targeting students who are at greatest risk.

In terms of media impacts, it is essential that people with mental illnesses be provided with up-to-date, accurate information about strategies for mitigating risk and knowing when to seek medical treatment for COVID-19 in a tailored and appropriate manner. This approach is vital because, as seen with children with OCD, information curated for the general public can instead overwhelm people with mental illnesses and lead to symptom deterioration [75]. Also, information should not just be about taking viral precautions. Information on maintaining healthy habits, including diet and physical activity, is vital.

Furthermore, despite the necessity of physical distancing strategies for mitigating the spread of COVID-19, this population also has an associated risk of loneliness and isolation. Thus, an interactive, continuous, and frequent monitoring and evaluation system must be developed for each patient to remotely assess their wellbeing and tailor treatment plans. This is important because psychological symptoms are shaped by patients’ unique personal and social contexts.

Lastly, targeted resource delivery approaches are required to reach marginalized groups, especially LGBTQ + communities that struggle with mental illnesses alongside other social barriers, including stigma. Social acceptance can be promoted through LGBTQ-affirming virtual extracurricular activities [85], social media resources such as the Trevor Project [65], and establishing protective spaces [86]. More importantly, the need for an intersectional approach for this population is non-negotiable, as some may be struggling with the double burden of sexual stigma and poverty, among others [87].

## 8. Areas of Future Research

Study [48] posed a paradoxical question as to whether the focus of public health interventions should be to maximize protective measures to limit the spread of COVID-19, with a consequence of increasing risk of psychosocial distress, or should public health do nothing and end up with an overwhelmed healthcare system, with a spike in deaths, because the goal is to protect children from potential psychological impacts of the COVID-19 pandemic? There is no simple answer, as both options have important and potentially irreversible implications. On the one hand, extended lockdowns exposed children to the risk of increased OCD symptoms, reinforced by economic stress on households and parents’ overprotective measures. On the other hand, reopening schools will not only expose children to risks of infection but the possibility of them infecting teachers, staff, and colleagues, which would also exacerbate adverse psychosocial outcomes [48,88,89].

Considering this theoretical impasse, we call for a more in-depth inquiry. A crucial finding of this review is that the mental health impacts of the pandemic are greater for school-aged children, potentially relating to their loss of daily routines. Longitudinal research is essential to determine long-term effects on pediatric mental health. Finally, additional research is needed to understand the mediating role of culture on the differential aggravation of OCD stress between different genders to provide better support to children.

## Figures and Tables

**Table 1:** Database search terms (MeSH terms were also used where applicable).

Component of the PEO Formulation	Search Strings
Population	“Children” OR “Kids” OR “Teens” OR “Teenagers” OR “Adolescents” OR “Youth”
Exposure	“Coronavirus” OR “COVID-19”
Outcome	“Obsessive-Compulsive Disorder” OR “OCD” OR “Hoarding” OR “Excoriation” OR “Trichotillomania” OR “Skin-picking disorder” OR “Tourette syndrome”

**Table 2:** Study findings: the effect of COVID-19 on OCD symptomology.

Theme	Factors	Article Mentions *n* (%)	Article References (Primary Author)
Individual level	Comorbidities, prior diagnosis, or pre-existing symptoms	10 (45.5)	[5,32,34,37,38,39,41,42,49,50]
	Age	4 (18.2)	[32,34,37,50]
	Gender	4 (18.2)	[31,38,48,50]
	Sexual orientation	1 (4.5)	[50]
	Race	1 (4.5)	[49]
	Pandemic origin fears and stress	3 (13.6)	[7,32,34]
	Isolation	2 (9.1)	[37,39]
	Educational attainment	1 (4.5)	[48]
	Personal beliefs and attitudes	1 (4.5)	[47]
Household level: relations and familial dynamics	Increased hygiene protocols	4 (18.2)	[31,32,36,38]
	Economic status (income and material deprivation)	4 (18.2)	[5,37,48,50]
	Diagnosis of relatives/family members	4 (18.2)	[4,34,36,49]
	Parental influence (beliefs, mental health status, pandemic worries, and practices)	3 (13.6)	[5,39,49]
Socio-structural level	Systemic factors/loss of services	3 (13.6)	[5,39,50]
	Social support	1 (4.5)	[46]
	COVID-19 media and preoccupation	2 (9.1)	[36,41]

## Data Availability

All data generated or analyzed during this study are included in this published article and its supplementary information files.

## Supplementary Materials

The following supporting information can be downloaded at: https://www.mdpi.com/article/10.3390/ijerph20237095/s1, Table S1: Summary of included articles. Table S2: Quality Assessment Score of selected articles.
