# Supplementary material for: The Parallel Pandemic: A Systematic Review on the Effects of the COVID-19 Pandemic on OCD among Children and Adolescents
Source: Int J Environ Res Public Health. 2023 Nov 22;20(23):7095. doi: 10.3390/ijerph20237095 (PMC10706205; doi:10.3390/ijerph20237095)
Supplement: Supplementary file 1 [file ijerph-20-07095-s001.zip › Summary of included articles.pdf]

Author	Year	Keywords	Study Design	Location	Age of pediatric sample	Mental health outcomes studied	Method of outcome assessment	Pandemic and OCD correlation	Key findings and conclusions
Fındık[44]	2023	Adolescent; Depression; Emergency; Mental health; Pandemic	Retrospective chart review	Turkey	Under 18	OCD	Prevalence	Positive correlation	OCD increased in the post pandemic period significantly.<br><br>There was no statistically significant difference between the years in terms of outpatient clinic appointment, transfer to hospitalization, reasons for presentation and psychiatric diagnoses of those transferred to hospitalization.
McKune[48]	2023	COVID-19; Pediatrics; Mental health; School-aged children; Anxiety, depression; Obsessive-compulsive disorder (OCD)	Longitudinal study (Composed of with three cross-sectional timepoints)	USA	Under 22	OCD	Prevalence	Positive correlation	The prevalence of anxiety, depression, or OCD moved from 47.1, to 57.2, to 42.2% across the three timepoints during the study.<br><br>By endline of the study, in April 2021, non-white children were at higher risk for depression and OCD.<br><br>Risk for anxiety, depression, and OCD was associated with students who lost a family member due to COVID-19 and who were identified as at-risk in previous timepoints.<br><br>Race was significantly associated with risk of depression and OCD, with Non-White participants being at higher risk of depression compared to White participants.<br><br>Parental belief that vaccinating their child was not important for the health of others in the community was protective against their child presenting at risk for OCD.
Stewart[49]	2023	COVID; Outcome; Age; Psychiatry; Access; Determinant; Child; Adolescent; Poverty; LGBTQ; Gender	Cohort study	Canada	Under 18	OCD	Prevalence, Worsening symptoms	Positive correlation	OCD Rate was multi-fold of those reported by pre-pandemic studies.<br><br>Across all age groups, the lifetime diagnosis of a psychiatric illness was a determinant for current psychiatric problems and/or unmet MH support needs during the pandemic.<br><br>Poverty, limited education, female and non-binary gender, xLGBTQ2S + status and special educational, psychiatric and medical conditions were differentially identified as determinants across age groups.
Tomczak[40]	2023	Tourette syndrome; Chronic tic disorder; Obsessive-compulsive disorder; Screen time; Children/adolescents; Pandemic	Cross-sectional study	USA	Under 18	OCD	Worsening symptoms	Positive Correlation	Children with OCD report a significant, subjectively worse impact of COVID-19 on home life as compared to children with tics and OCD + Tics.<br><br>Children with OCD, CTD, and OCD + Tics reported that the pandemic had a negative impact on

									activities outside of school, relationships with friends, and family/ community gatherings.
Charalampopoulou[5]	2022	Autism; Children; COVID; Mental health; Pandemic.	Cross-sectional study	Canada	Under 18	OCD	Worsening symptoms	Positive correlation	In Autistic children whose MH deteriorated, OCD symptoms were significantly higher than in unchanged children.
Hamid[4]	2022	Pediatrics; Obsessive compulsive disorder; Anxiety; Depression; Covid-19	Cross-sectional study	Canada	Under 18	OCD	Prevalence	Positive correlation	15.5% of participants exhibited OCD symptoms.<br><br>Significant association between diagnosis of family member or frontline worker and presence of OCD symptoms.
Henein[32]	2022	Obsessive–compulsive disorder; Children and adolescents; COVID-19; CAMHS referrals; Symptom Exacerbation; Cognitive behavioural therapy; Pharmacotherapy; Service evaluation	Retrospective chart review	UK	Under 18	OCD	Prevalence, Worsening symptoms	Positive correlation	5.62% assessments to Child and Adolescent Mental Health Services were related to OCD in 2020, which is higher when compared to 1.30% and 2.27% assessments in the pre-pandemic times (2018 and 2019).<br><br>Adolescent Mental Health Service (CAMHS) fell in 2020, whilst the proportion of assessments related to OCD increased significantly
Ibeziako[37]	2022	Coronavirus pandemic; Diagnosis; Psychiatric; Eating disorders; Ed boarding; Mental health; Pandemics; Trend; Inpatients; Depressive disorders; psychiatry	Retrospective chart review	USA	Under 18	OCD	Prevalence	Positive correlation	Trend of increased hand washing and other infection control compulsions did not increase during larger second wave
Khan[7]	2022	COVID-19; Pandemic fears; Adolescents; Mental disorder; Obsessive-compulsive symptoms	Cross-sectional study	Qatar	Under 18	OCD	Prevalence	Positive correlation	Over half of the young People with mental health disorders that had significant pandemic over half seemed to have significant Obsessive-Compulsive symptoms<br><br>pandemic fear likely to be associated with OCD
Kroon[38]	2022	COVID-19; OCD; Mental health; Family; Children	Longitudinal study	Australia	Under 18	OCD	Worsening symptoms	Positive correlation	Overall sample showed increased OCD symptom severity compared to normative pre pandemic sample.<br><br>There was a significant positive association between parental worries and child mental health symptoms.
Tanir[35]	2022	Not provided	Cross-sectional study	Turkey	Under 18	OCD	Worsening symptoms	Positive correlation	Significant increase in contamination obsessions and cleaning/ washing compulsions.<br><br>There was a significant relationship between the change in CY-BOCS scores with talking about COVID-19 and diagnosis of someone familiar.

Tekin[39]	2022	COVID-19 pandemic; Psychosocial symptoms; Adolescent; Quality of life	Cross-sectional (With retrospective data collection)	Turkey	Under 18	OCD	Prevalence	Positive correlation	A statistically significant increase was observed in all depression, anxiety, and OCD scores evaluated with the RCADS-parent report compared with the pre-pandemic period.
Carmi[29]	2021	OCD; CBT; COVID-19	Cohort study	Israel	Under 22	OCD	Worsening symptoms	No correlation	OCD patients did not experience exacerbated symptoms during COVID-19 at their 2- and 6-month follow-ups.
Halil1[31]	2021	Obsessive-compulsive disorder; Covid-19; Stressör; Children	Longitudinal study	Turkey	Under 18	OCD	Worsening symptoms	Positive correlation	OCD severity was significantly increased during the pandemic.<br><br>sexual obsessions increased as well as contamination and illness obsessions.
McKune[47]	2021	COVID-19; Mental health; Psychosocial impacts; Vulnerable population; Pandemic	Cross-sectional study	USA	Under 22	OCD	Worsening symptoms	Positive correlation	Loss of household income, being female and lower school grade all associated with increased OCD symptoms stemming from Pandemic.
Schwartz-Lifshitz[50]	2021	COVID-19; OCD; Exacerbation; Course	Longitudinal study	Israel	Under 18	OCD	Worsening symptoms	No correlation	OCD symptoms did not exacerbate.<br><br>More patients improved then deteriorated
Tombeau-Cost[36]	2021	COVID-19; Child and adolescent psychiatry; Neurodevelopmental disorders	Cross-sectional study	Canada	Under 18	OCD	Worsening symptoms	Mixed findings	obsessions/compulsions deterioration was correlated with having a pre-diagnosed psychiatric disorder, greater economic concerns and stress from social isolation.<br><br>Deterioration highest in ages 13-18
Conte[45]	2020	Tourette syndrome; COVID-19; Pandemic; Mental health; Tics; Children; Adolescents	Cross-sectional study	Italy	Under 22	Tourette's, OCD	Prevalence, Worsening symptoms	Positive correlation	67% of individuals with TS were rated by their parents as having a worse clinical condition.<br><br>The most worsened symptoms included tics, hyperactivity, rage attacks, obsessions/compulsions, and anxiety.
Darvishi[30]	2020	Cognitive errors; Obsessive-compulsive disorders; Young people; Coronavirus disease 2019	Cross-sectional study	Iran	Under 22	Cognitive Errors, OCD	Worsening symptoms	Mixed findings	67.3% of subjects evaluated exhibited OCD symptomology, with significant differences in gender as well as cognitive errors.
Nissen[33]	2020	Obsessive compulsive disorder; Children; Adolescents, COVID-19, Trauma, OCD	Case-Control Study	Denmark	Under 22	OCD	Worsening symptoms	Positive correlation	Worsening of OCD symptoms occurred for both newly diagnosed and previously treated patients during the pandmic.<br><br>Aggressive symptoms, early age of onset and familial ADHD predicted worsening
O'Dor[41]	2020	Obsessive-compulsive disorder; Pediatric; Psychiatry; PANS; PANDAS; Tics	Cross-sectional study	USA	Under 18	OCD	Prevalence, Worsening symptoms	Positive correlation	Children with suspected or diagnosed COVID-19 experienced new or worsened psychiatric

									symptoms, particularly mood lability, OCD, and anxiety.<br><br>Caregivers reported negative impacts on their child's relationships with friends, extended family, participation in hobbies, and academic skills.<br><br>However, the majority of respondents felt the pandemic had a positive impact on their relationship with their child
Oosterhoff[46]	2020	Not provided	Cross-sectional study	USA	Under 22	Hoarding	Other	Mixed findings	19.7% of adolescents exhibited hoarding behaviours.<br><br>Positive association with females and news monitoring.

Summary of articles (n= 22)
